# Experiments on the Analysis of Atmospherical Air; with Observations on Dr. Priestley's Experiments, Lately Read before the American Philosophical Society

**Published:** 1799-03

**Authors:** 


### *Experiments on the Analyſis of Atmoſpherical Air; with Obſervations on Dr.* Priestley's *Experiments, lately read before the American Philoſophical Society.*

THE ſyſtem of Lavoisier ſeems now ſo firmly eſtabliſhed, as to ſtand in need of no formal vindication; the converſion of ſeveral eminent and ſtrenuous advocates for the phlogiſtic doctrine, to this ſimple and elegant theory, has within theſe few years contributed not a little to its ſtability; yet, whilſt a philoſopher ſo diſtinguiſhed in the annals of ſcience as Dr. Priestley, ſtill maintains the contrary opinion, it is certainly worth while to examine, upon what grounds he defends a ſyſtem, now almoſt univerſally exploded.

Our atmoſphere, or the air which we breathe, is compoſed of two permantly elaſtic fluids, of very different and oppoſite qualities; the one unfit for reſpiration or combuſtion, and therefore called azote, or azotic gas, the other poſſeſſing both theſe properties in a very eminent degree, and on that account denominated *pure* or *vital* air, or more juſtly, and in conformity to the new nomenclature, oxigen-gas

From the intimate combination of theſe two aeriform fluids, in proportions which we ſhall ſtate hereafter, reſults what we in general term common or atmoſpherical air.

Modern chemiſtry furniſhes us with abundant means of determining the nature and relative proportions of theſe two gaſes, which, not being chemically united, but exiſting merely in a ſtate of intimate mixture or combination, are eaſily ſeparable by a ſubſtance poſſeſſing an elective attraction for either of them.—Thus, if pure mercury be expoſed for ſome time in cloſed veſſels filled with atmoſpherical air to a degree of heat nearly approaching to ebullition, a quantity of air will diſappear, and the mercury be converted into a red powder, which has gained an addition of weight equal to that of the air abſorbed. On examining the air in the veſſels, we ſhall shall find it differing materially in properties from that which we began to experiment upon. A candle plunged in it is immediately extinguished, and a live animal introduced beneath a bell-glass filled with it, perishes instantaneously: in short, it appears to have lost all power of supporting either respiration or combustion. If the red powder formed during the process, be next submitted to examination, and exposed to a red heat in a retort, to which is adapted an apparatus for collecting such gaseous fluids as may be produced, a quantity of air will be obtained precisely equal in bulk to that which had before disappeared, at the same time that the red powder rises in vapour, and is collected in the receiver, in the form of running mercury. On subjecting this new aerial product to the same trial as the former, we shall find that, instead of extinguishing flame and destroying life, it is eminently capable of supporting both. A taper immersed in it burns with a dazzling splendor; and red hot charcoal introduced into it, instead of consuming quickly, as in atmospherical air, burns with great rapidity, attended with a decrepitating noise and unusual production of heat and light.

It appears therefore from these experiments, that mercury, at a degree of heat approaching to ebullition, possesses the power of decomposing atmospherical air, and of combining with the pure part or oxygenous gas, which is thus separated from the other constituent part, or azote, and therefore furnishes us with a mode of determining their respective natures. To ascertain exactly the proportions in which they are combined, other means must be made use of, as in these experiments the mercury is never able entirely to separate the last portions of oxygen, partly on account of its attraction for the principle of heat, and partly on account of the mutual attraction which subsists between the two component parts of the atmosphere.

If a bell-glass be inverted over a solution of liver of sulphur, the air contained therein will suffer a dimunition, which will go on during several days, and then cease. The unabsorbed part will be found to be pure azote, the oxygen alone being absorbed, and the diminution, when accurately examined, will be found to be about 27 parts in 100. So that atmospheric air is a combination of azote with oxygen gas, in the proportion of about seventy-three parts of the former to twenty-seven of the latter.

It is evident that these proportions must somewhat vary in certain situations, and be liable to alteration from local changes in the atmosphere* ; yet the difference is never very great, generally not more than three or four parts in a hundred ; and if we mix in the proportions here given, a quantity of azote obtained by means of liver of sulphur, with the gas expelled from the red

* In a future number we shall give the result of various expeeriments made on the atmosphere of London and its vicinity.

red calx of mercury, a precipitate *per se*, we ſhall obtain a ſpecies of air reſembling atmoſpheric or common air in every reſpect, being capable, like it, of ſupporting life and combuſtion, and affording, when ſubmitted to analyſis, preciſely ſimilar reſults.

We ſhall now advert to thoſe experiments of Dr. Prieſtley, which have given riſe to theſe obſervations, and which were inſtituted with a view of controverting the preceding ſtatement. In all caſes of what is called the phlogiſtication of air, there is, ſays Dr. Prieſtley, an emiſſion of ſomething from the ſubſtance, which the antiphlogiſtians ſuppoſe to act by ſimple abſorption. This is the ſame that has been called phlogiſton, or the principle of inflammability, which, uniting with dephlogiſticated air, forms with it part of the azote found after the proceſs.

The experiments adduced in ſupport of this opinion, and upon which he lays the greateſt ſtreſs, are made with *charred bones*, heated in atmoſpherical air; and as nothing in them is volatile, except that which conſtitutes their blackneſs, I thought, ſays Dr. Prieſtley, that they would be a convenient ſubſtance wherewith to make theſe experiments.

We ſhall extract a few of them from his paper, and then proceed with our remarks. "Having by means of a burning lens heated 140.5 grains of well-burned black bones in 28.75 ounce meaſures of air, it was reduced to 20 ounce meaſures completely phlogiſticated, without any mixture of fixed or inflammable air in it. According to this experiment, the quantity of pure air in 100 ounce meaſures of atmoſpheric air, was only 15.78 parts inſtead of 27.

"Heating 267 grains of theſe bones in 30 ounce meaſures of air, it was reduced to 25.5 ounce meaſures completely phlogiſticated, which was in the proportion of 15 parts dephlogiſticated air in 100 of atmoſpherical. In theſe experiments with bones there is ſometimes a ſmall *loſs of weight*, owing, I doubt not, to ſomething beſides phlogiſton being expelled from them by the heat of the lens &c. I had ſimilar reſults from experiments made with ſmall poliſhed *ſteel needles*, for when they were heated ſo as only to become blue and were not melted they gained very little if any weight, and diminiſhed the air only in about the ſame proportion as the bones.

"I heated 200 grains of poliſhed needles in 16.75 ounce meaſures of air, when it was redneed to 13.5 meaſures completely phlogiſticated without any mixture of fixed or inflammable air in it; ſo that the diminution was in the proportion of 19.4 parts in 100.

"There was not however the ſame certainty in the experiments with the needles, and ſtill leſs with the iron, as in thoſe with the bones. *They generally gained a little weight, and diminiſhed the air more than the bones.*

"When

"When the needles were heated over lime water, a thick cruft was formed upon it, but there was not fuch a precipitation of the lime as in the experiments with the bones."

It is hardly neceffary to have recourfe to experiment, to prove the inaccuracy of the preceding obfervations. In all the cafes of diminution of the air by the black bones, a quantity of carbonic acid was produced by the union of the carbon contained in them with the oxigen of the atmofpherical air. The lofs in the weight of the bones, and the precipitation that took place when the experiments were made over lime water proves this fully, and it is probable, that in examining the refiduum, Dr. Prieftley was not fufficiently careful to feparate this gas from the phlogifticated air that remained, which would confequently efcape detection, if he made ufe of the nitrous air as a teft of its purity. This opinion is ftrengthened by the various refults he obtained in his experiments; fometimes, though the air was completely phlogifticated, the dimunition was only 15 parts in 100; at other times 15.75, and once 19.4.

The experiments with fteel needles are not more conclufive, or lefs liable to objection. Carbon is one of the conftituent parts of fteel, and it is from this fource that we muft derive the carbonic acid, formed when they are heated. Thus, while one portion of the oxigen of the atmofpherical air united with the carbon of the fteel, to form carbonic acid, which was in part abforbed by the water, another portion united with the iron, which was by that means partially oxydated, and received a fmall addition of weight. The phlogifticated air found after the procefs is what exifted originally in the atmofpherical air employed.

But, fays Dr. Prieftley in his experiments, there is always a greater proportion of phlogifticated air found after the procefs, than enters, according to the anti-phlogiftians, into the compofition of atmofpherical air. This additional quantity, he fuppofes, is derived from the union of part of the oxigen gas of the atmofpherical air, with the phlogifton emitted from the bones, a fuppofition, which, however, we hope the following experiment will completely refute.

We expofed a piece of black bone (which had been charred in an intenfe degree of heat) to the focus of a burning lens in a bell-glafs, placed on a quickfilver bath, and half filled with pure oxigen gas, expelled by gentle heat from oxigenated muriate of potafh; the volume of the air was increafed by the application of heat, and after it had been fuffered to cool, appeared fomewhat augmented. Having withdrawn the bone that was unconfumed, a folution of cauftic alkali was introduced under the bell-glafs, which abforbed nearly nine-tenths of the whole gas, which therefore confifted of carbonic acid. The remainder, on examination, was pure oxigen gas, with the exception exception of a small bubble of azote, which we are convinced existed in the gas previous to its being introduced under the bell-glass. After such unequivocal proof, that when phlogisticated air is not employed, none is produced, it is certainly presumptive, that it is not produced in the other instances; and, indeed, it appears strange to us that the union of what is termed phlogiston with oxigen, should at one time generate fixed air, and at another azote, without any apparent cause for such a capricious result.

The observations which Dr. Priestley makes on the phlogistication of nitrous acid, by absorbing nitrous gas, are not a little extraordinary. They seem almost to prove, that he still does not comprehend the principles of the new theory:

" That the phlogistication of nitrous acid is owing, in some cases, to its *imbibing* something, and not always to its parting with any thing which the antiphlogistians maintain, is evident from its becoming phlogisticated by *imbibing nitrous air*. M. Fourcroy supposes, in his " *Philosophie Chymique*", that the conversion of the common nitrous acid into the phlogisticated, is always ocasioned by its parting with oxigen. That this is sometimes the case, I have demonstrated in my experiments, with heating it in long glass tubes, but in the present case it is not possible, that the acid should have parted with any thing, and least of all with oxigen, since the small residuum of nitrous air is pure azote".

Let us examine into the constituent principles of nitrous acid, and see if we cannot prove this strange paradox true, that nitrous acid, by imbibing nitrous air, loses oxigen. Azote combined with oxigen, nearly in the proportion of one to two by weight, forms nitrous gas, which is by no means saturated with oxigen, since in this state it seizes it with avidity from the atmosphere. The further addition of oxigen converts it into a powerful acid. When the proportions by weight of oxigen and azote, says Lavosier, are below three parts of the former to one of the latter, the acid is red-coloured, and emits copious fumes. In this state, by the application of gentle heat, it gives out nitrous gas, and we term it in this degree of oxigenation, *nitrous acid*. When four parts by weight of oxigen are combined with one of axote, the acid is clear and colourless, more fixed in the fire than the nitrous acid, has less odour, and its constituent elements are more firmly united: this species of acid, in conformity with our nomenclature, is called nitric acid. Thus *nitric acid* is the acid of nitre surcharged with oxigen; *nitrous acid* is the acid of nitre surcharged with *azote*, or what is the same thing, *nitrous gas*, and this latter is azote, not sufficiently saturated with oxigen to possess the properties of an acid.

Nothing can be more easily explained, according to these principles, than the phlogistication of nitrous acid, by imbibing nitrous air. Its proportion of of azote becomes thereby increaſed, a proceſs which is equivalent to the ſubtraction of a portion of the other conſtituent part, or oxygen; and thus confirms the accuracy of M. Fourcroy in ſtating, that the phlogiſtication of nitrous acid is always occaſioned by its parting with oxygen.

The other experiments detailed in this paper are what have been repeatedly before urged, and as often refuted. Dr. Prieſtley has himſelf proved, that air confined in wet bladders is externally affected; beſides, the leaſt tendency to putrefaction would materially affect the reſult of the experiment. With regard to the formation of phlogiſticated air, by keeping long confined in a bottle a mixture of dephlogiſticated and inflammable airs, we can only affirm, that with us it never would ſucceed. A mixture of theſe two airs, confined over mercury near two months, ſuffered no perceptible change; and another mixture, kept three months, was diminiſhed about one fortieth of its original bulk; but both exploded with a degree of violence that did not appear to us at all inferior to that of newly prepared gaſes; and being accurately examined, did not afford the leaſt portion of phlogiſticated air. We ſhall conclude with obſerving, that theſe experiments of Dr. Prieſtley have not, upon the whole, that ſcrupulous exactitude, which in philoſophical inveſtigations we have a right to demand, and particularly ſo, in experiments inſtituted with a view of eſtabliſhing a controverted doctrine.

Much as we admire the genius and talents of Dr. Prieſtley, we cannot help thinking, that his rejection of the antiphlogiſtic doctrine ariſes not ſolely from want of demonſtrative experiments. It is difficult for a man to abandon opinions which he has been the firſt to promulgate—opinions founded on a ſeries of diſcoveries the moſt brilliant, perhaps, ever made by one individual in the ſcience of chemiſtry. Yet from Dr. Prieſtley we can expect every thing; a ſlight acquaintance with his works will convince any one, that he is not the ſlave of hypotheſis; and that, influenced by facts, he has more than once recanted his own opinions, and we are not without hopes yet of hearing him join with the reſt of the philoſophic world, in adopting the ſublime truths of the unfortunate, but immortal Lavoiſier.

*London, Feb.* 6, 1799. T.

